# A Compendious History of Small-Pox, with an Account of a Mode of Local Treatment Which Prevents Scarring

**Published:** 1834-01-01

**Authors:** 


A Compendious History of Small-Pox; with an Account of a Mode of Local Treatment which prevents the Seaming or Scarring of the Skin, and the Occurrence of that Aggravation of Symptoms in the advanced Stages of the Disease hitherto denominated Secondary Fever. By *Henry George*, Surgeon, Surgeon Extraordinary to H. R. H. the Duke of Gloucester. Octavo, pp. 112. Sept. 1833.

A history, however compendious, or however learned, of the small-pox, at this time of day, could scarcely be contemplated by any member of the profession, were there not improvements in the treatment or prevention introduced, to secure public attention. Yet Mr. George has contrived to make a short historical sketch of small-pox, however trite the subject, considerably interesting, while he has shewn that he was capable of searching ancient writings and records to which few modern authors have recourse. On this part of the book, however, we cannot dwell, since the sketch itself is a kind of condensed analysis.

Under the head of treatment, Mr. George remarks that, considering the innumerable suppurating processes going on in the skin, during confluent variola, and the extensive chain of sympathies between the surface and the internal organs, the wonder is, that the mortality is not even greater than it actually is. Our author is induced to doubt whether the tumult or ebullition in the constitution, under small-pox, is not referrible to nervous irritation rather than high vascular action. Fordyce had similar doubts, and was anxious to separate from the class pyrexia, those sympathetic or symptomatic fevers depending on local inflammations, among which he reckoned variola, as the following passage from his works shews.

> "In the small-pox, if the infectious matter be applied to a wound, an inflammation is produced in that wound, in consequence of which a fever arises; if the poison of a bee be infused into a wound made by the sting of the animal, or if the poison of any other animal be injected into a wound by its sting or tooth, an inflammation arises in the part where the wound is made, and that inflammation produces affection of the whole system, some of the symptoms of which may be similar to fever, but are not the disease intended to be described here by that name. It might happen that a great inflammation might be immediately produced in a wound into which variolous matter was infused, that such an inflammation may produce affection of the whole system in a day or two afterwards; yet that affection is by no means to be called fever, which takes place, being only induced after the suppuration of the wound is complete, which is on the seventh or eighth day. It is also to be observed that when, in consequence of a fever, produced by infectious matter, some topical inflammation arises, and the fever is carried off by it, that such topical inflammation as in the small-pox produces affection of the system, in which some of the appearances are similar to some of the appearances which take place in fever: such affection of the system has frequently been called fever. In the small-pox, for instance, such affection has been called secondary fever, although it in no way has any thing of the essence of the disease." 58.

Many authors and practitioners consider the secondary fever as a kind of essential one, requiring the usual depletive measures; but the best medical practitioners regard the secondary variolous fever as of the irritative class, analogous to that from a poisoned wound—or from any wound in a bad constitution. One of the distinctions which Mr. G. draws between small-pox fever and other fevers, is the non-suppression of secretion in the former.

"From my own experience I would venture to assert, that in the different stages of small-pox, none of these appearances (non-secretion) are to be observed. The kidneys (if we do not treat the disease as febrile) act plentifully, the bowels require very trifling assistance to procure the most satisfactory relief, and the appetite, depending of course on the secretion of the gastric fluid, becomes almost voracious, if our medicated remedies in the early stages of the disease are selected with the view of tranquillizing the system, and at the same time sustaining its powers. I have already expressed a belief that the formidable symptoms in the advanced stages of this disease are partly to be attributed to our own mismanagement at its commencement. One important consequence of strictly treating this malady as fever in its early stages, is to deprive the patient of this very appetite. This effort of nature to support him under the severest of trials—this resource is totally or partially subdued by our very remedies, and the nourishment which is permitted in no way tends to mitigate the evils; the stomach is well known to possess a power almost magical over every portion of the body: the state of discontent above-mentioned aggravates the constitutional disturbance, and a series of diseased actions in the latter stages of this malady are, by the advocates for the existence of fever, again aggravated by the employment of the very remedies which have in some degree contributed to produce them. If we have not fever to contend with, I believe that rule of conduct would be most excellent, which would seriously warn us to beware of offending this organ (the stomach.)" 65.

This, we confess, is a startling doctrine, and, as Jonathan says, "requires confirmation." We have no hesitation, however, in saying, that we have seen very injurious consequences result from treating the eruptive fever of this and other specific diseases, by active depletion. The constitution is evidently labouring to throw out a poison upon the surface of the body—and that labour assumes the form of an inflammatory fever. If the powers of life be interrupted (they occasionally require control) the eruptive process will be incomplete, and much mischief will be incurred. The worst of it is, that variola is very often mistaken for some other complaint till the eruption appears, when it is sometimes too late to retrace false steps. The best way is to watch carefully, and rather moderate than check the constitutional symptoms.

The local treatment which our author has found beneficial, was pointed out about two or three years ago, in the Medical Gazette, the sum and substance of which are contained in the following quotation.

"The treatment consists in covering the body as completely as possible with any absorbent powder, (I have generally used the calamine;) the advantages which follow the use of this dressing in the early stage of this disease, are, to moderate the violence of the local inflammations, and to prevent the painful tumefaction of the common integuments. After the calamine has been applied some hours, a very sensible difference is to be observed in the appearance of the parts so covered; the areola of each pustule being much less distinctly marked. It is not unreasonable to suppose, that even at this moment some advantage is gained by the application; the quantity of pus secreted may by this circumstance be diminished, and a great saving made of the powers of the constitution; but it is in the advanced stages of this disease that the greatest benefit is derived from this method of local treatment, particularly at that period which Sir Henry Halford, in his Essays and Orations, points out as being eminently critical. In the essay entitled, 'On the Necessity of Caution in the Estimation of Symptoms in the Last Stages of some Diseases,' it is observed, that 'The physician may fairly acquiesce in the fears of a family, when, on the completion of the eruption, he sees the face and breast one mass of disease, and may most reasonably doubt the capability of the constitution to maturate and perfect so large an eruption. But he must not hold out unfounded hopes to the parents, if the malady proceeds in the next stage, in a most satisfactory manner, beyond his expectations; the pustules ripening fully, and the process being complete; for, alas! at this very moment, it may be, the patient is sinking—is dead. The powers of his constitution being exhausted by the efforts it has made, and no longer equal to the accomplishment of a protracted cure.' At this crisis, too, in addition to the source of danger above-mentioned, might well be added another of the greatest magnitude; viz. those large portions of exposed cutis which about this time are torturing the sufferer beyond endurance, rendering his situation so truly horrible that to exclaim—

Dî meliora piis, erroremque hostibus illum, would be almost justifiable.

As I said before, it is at this moment we have it in our power not only greatly to circumscribe the field of suppuration, but to heal, on destroying the cuticle, by the process recommended, every pustule on the body, almost in the space of a few hours. At such a crisis what an immeasurable advantage is gained by the exercise of such a power! the drain on the constitution may thus at our discretion be partially or completely closed. To accomplish this, is indeed, as I have experienced, a disgusting and painful task; but what difficulties will not with alacrity be encountered by the hand of duty or of friendship. By the same process, those extensive portions of exposed cutis may be rapidly healed; exclude them also from communication with the atmosphere, and they soon cease to be sources of irritation. I have seen patches of exposed cutis, six or eight inches in diameter, at the end of two or three days, by this treatment, no longer occasioning disturbance. If no other benefit followed than the mere relief from pain, great must still, by every feeling mind, be thought the advantage; for it is impossible to imagine the agonizing sufferings of the patient, from this circumstance. I have often seen every nerve of the body quivering with distress on the slightest exertion, and have as often witnessed the firmest mind sinking into a state of almost childish weakness, from the extremity of the sufferings." 74.

Fifteen or twenty years ago it would have been thought useless to say any thing about the nature or treatment of variola, since we were then considered to be in possession of an infallible preventive, or at least substitute, for that disgusting and disfiguring malady—in the mild disorder, VACCINIA. But it is not to be disguised that TIME has greatly diminished the protective powers of vaccination; and we see so many instances of subsequent variola, and that of a very violent kind, that we greatly doubt whether, if every lustrum or decade continues, in the same ratio, to exhibit failure, the public or the profession will, one hundred years hence, know anything of cow-pox, but from history!! We have been strenuous advocates for vaccination, and still continue to recommend it, in preference to inoculation, when our opinions are asked; but we should be very loth to urge it on parents in the same energetic manner that we did fifteen years ago. To represent vaccination as an infallible, or almost infallible preventive of small-pox, is more than we would do—and we apprehend that it is more than is justifiable by any conscientious practitioner. There is one evil (among some thousands of others) to which humanity is liable, from the moment that man leaves his cradle—VARIOLA. Of the two preventives or substitutes, inoculation is the more certain, but the more severe—vaccination the more easy (free, indeed, from suffering), but the less powerful protector. If this estimate be not correct, it is at least honest.

